# Antioxidant Peptides from Tiger Nut (*Cyperus esculentus* L.): Chemical Analysis and Cytoprotective Functions on HepG2 and Caco-2 Cells

**DOI:** 10.3390/foods14030349

**Published:** 2025-01-22

**Authors:** Yu Quan, Lin Chen, Meiqi Fan, Xia Zhao, Jianxiong Hao

**Affiliations:** College of Food Science and Biology, Hebei University of Science and Technology, Shijiazhuang 050018, China; 13893423105@163.com (Y.Q.); c2537419491@163.com (L.C.); 15833986221@163.com (M.F.)

**Keywords:** tiger nut, peptide, oxidative damage, cytoprotective effect, antioxidant activity

## Abstract

Tiger nuts were enzymatically hydrolyzed by Alcalase and then separated and purified by ultrafiltration classification and Sephadex G-15 fractionation to obtain tiger nut peptides. Their chemical antioxidant activities and cytoprotective functions on HepG2 and Caco-2 cells were systematically evaluated in this study. The tiger nut peptides (TNP) were found to perform excellent antioxidant activity supported by their chemical and cell antioxidant behaviors, amino acid composition, and morphological observation. Higher 1,1-diphenyl-2-picrylhydrazyl radical scavenging activity (DPPH• RSA, 64.05–124.07%) and ferric ion-reducing antioxidant power (FRAP, 0.17–1.78 μmol/mL) were observed in the TNP with more hydrophobic amino acids (41.77 ± 1.36 g/100 g) compared with traditional soybean and peanut peptides. Furthermore, the peptides from tiger nut (TNP, TNP-4, T1, T2, T3) could effectively protect H_2_O_2_-induced HepG2 and Caco-2 cells from oxidative damage by enhancing endogenous antioxidant enzyme activities and reducing oxidative stress levels, especially the T3 peptides purified from the fraction less than 1 kDa molecular weight. The catalase, superoxide dismutase, and glutathione peroxidase activities significantly increased, and the contents of intracellular reactive oxygen species and malondialdehyde decreased. This study highlights the potential of the peptides from tiger nuts as antioxidant ingredients for food applications.

## 1. Introduction

Normal physiological metabolism is invariably accompanied by the transition from inhaled oxygen into reactive oxygen species (ROS). The ROS level can increase in the body under an unhealthy external environment and/or abnormal body metabolism [1]. Oxidative stress by surplus ROS causes cellular oxidative damage, which has been a significant risk factor for chronic illnesses, such as cardiovascular disease and diabetes [2]. To mitigate the negative impact, antioxidants are developed to capture and neutralize the free radicals. However, the current synthetic antioxidants in food are subjected to strict supervision due to safety, high cost, and limited activities [3,4]. Being well-tolerated and safe, some bioactive peptides, such as goat milk casein peptide, wheat germ peptide, camel bone peptide, scallop peptide, and hydrilla verticillata peptide [3,5,6,7,8], have been demonstrated to be capable of scavenging free radicals and reducing peroxidation.

Antioxidant peptides are oligopeptides or polypeptides with antioxidant properties, as a kind of bioactive peptide [9]. They can perform biomolecular synergistic effects when the equilibrium between intracellular oxidant production and antioxidant content is compromised, exhibiting electronic provisioning capability, chelating metal ions, and scavenging excessive free radicals [10]. Antioxidant peptides have received significant attention in recent years because of their great potency, natural safety, and efficient absorption [11,12]. Among the existing reports of antioxidant peptides derived from legume and nut sources, the antioxidant peptides from soybeans have been identified to be effective in scavenging superoxide radicals and protecting cells [13]. And the amygdalin hydrolysate from natural and conjugated enzymes [14], the peptides (Thr-Pro-Ala, Ile/Leu-Pro-Ser, and Ser-Pro) from peanuts [15], and the peptides from tiger nut [16] also performed potential antioxidant activities. These emphasize the potential of antioxidant peptides from legume and nut sources as promising candidates for food additive and nutraceutical applications. In general, the peptides derived from hydrolyzed proteins possess much greater antioxidant activity compared to their parent proteins [17]. The antioxidant capacities of peptides can be further affected by various factors, including their molecular weight and amino acid composition [3,18,19].

Tiger nut (*Cyperus esculentus* L.) is a tuber of the sedge family, widely cultivated in Europe, Africa, and Asia [20]. It is rich in vital nutrients and bioactive molecules including fats, proteins, vitamins, and phenols, and is seen as a valuable material for the food industry. Most of the tiger nut studies focus on the extraction or application of its oil, but the defatted tiger nut contributes to significant waste [21]. The increasing global population and living standards have led to increased demand for quality protein products. And there is a heightened emphasis on the creation and utilization of new plant protein resources. It is noted that the protein from defatted tiger nut has a high content of essential amino acids (AAs) (46.03%), beyond the WHO/FAO standard of 36%, and is greater than soybean protein (41%) [22]. Moreover, tiger nut protein has been reported to facilitate blood circulation and immune regulation, and reduce cardiovascular disease risk [23,24]. Tiger nut is expected to be a promising and underutilized source of available excellent proteins and/or bioactive peptides.

Currently, research on tiger nuts has primarily focused on extraction optimization and the properties of protein, few studies were further conducted on the function properties of its peptides [22,25]. Direct insights into the integral processes of intracellular absorption and substance metabolism are provided by cell experiments. Combining the strengths of chemical and cell experiments yields a comprehensive and accurate assessment of the antioxidative potential of peptides. In this work, peptides were obtained from tiger nut by enzymatic hydrolysis, ultrafiltration classification, and Sephadex G-15 fractionation, and their antioxidant activities were further investigated by a combined analysis of chemical antioxidant behavior, amino acid composition, cellular antioxidant properties on H_2_O_2_-mediated oxidative-injured HepG2 and Caco-2 cells, and morphological observation for the first time. This study could provide more insights and guidance for the development and potential application of tiger nut peptides.

## 2. Materials and Methods

### 2.1. Materials

Tiger nuts were obtained from a local market in Hengshui City (Hebei, China). Alcalase (200 U/mg) was purchased from Shanghai Yuanye Biotechnology Co., Ltd. (Shanghai, China). DPPH• was purchased from Shanghai Macklin Biochemical Technology Co., Ltd. (Shanghai, China). ABTS•^+^ and MTT kit were purchased from Beijing Solarbio Technology Co., Ltd. (Beijing, China). HepG2 cells, Caco-2 cells, FBS (10% [*v*/*v*]), and MEM were purchased from Changsha Abiowell Biotechnology Co., Ltd. (Changsha, China). Penicillin–streptomycin (1% [*v*/*v*]) was purchased from Shanghai Beyotime Biotechnology Co., Ltd. (Shanghai, China). Other chemicals used were analytical grade.

### 2.2. Preparation and Experimental Optimization of TNP

#### Preparation of TNP

TNP was prepared from tiger nuts according to the method described by Alemán et al. [26] with modification and we used the previous laboratory results [16]. Briefly, the defatted tiger nut meal was filtered through a 150 μm standard sieve, combined in a ratio of 1:6 (powder/solution, *w*/*v*) with deionized water, and the protein solubility was determined using the Coomassie Brilliant Blue between pH 7.0 and 11.0. The pH was determined to be 9.0 in conjunction with the enzymatic conditions for Alcalase. The reaction conditions were set as 10,000 U/g of enzyme/substrate ratio, 3 h of hydrolysis time, and 55 °C of hydrolysis temperature. Hydrolysis was then carried out and the mixture was centrifuged by a centrifuge (HC-3018, Anhui USTC Zonkia, China) at 7156× *g* for 20 min at 25 °C to collect the aqueous phase, which was freeze-dried.

The degree of hydrolysis (DH) of TNP was calculated by Equations (1) and (2). The initial pH of enzymatic hydrolyzed was 9.0 [27].

(1) $$\mathrm{DH}\;\left(\%\right)=\frac{V\times C}{M_{P}\times H_{\mathrm{tot}}\times \alpha }\times 100\%$$

(2) $$\alpha =\frac{10^{\mathrm{pH}-\mathrm{pK}}}{10^{\mathrm{pH}-\mathrm{pK}}+1}$$

where *V* is the NaOH volume (mL), *C* is the concentration of NaOH (mol/L), *M_P_* is the mass (g) of the protein (N × 6.25), *α* means the average level of amino group dissociation; *H_tot_* means the number of peptide bonds per gram of protein substrate (mmol), and *pK* was determined using the 7.0.

The DPPH• radical scavenging activity (RSA) was calculated by Equation (3) [28].

(3) $$\mathrm{DPPH}•\;\mathrm{RSA}\;(\%)=(1-\frac{A_{1}-A_{2}}{A_{0}})\times 100$$

where *A*_1_ represents the value of samples, *A*_2_ represents the value of samples mixed with absolute ethanol, and *A*_0_ represents the value of DPPH• solution mixed with absolute ethanol.

### 2.3. Basic Functional Properties

#### 2.3.1. Preparation of Tiger Nut Protein

The tiger nut meal (100 mesh) was dispersed with deionized water with pH adjusted to 9.0 at a ratio of 1:8 (powder: solution, *w*/*v*). The obtained mixture was stirred by a magnetic stirrer (DF-101S, Gongyi Yuhua, China) at 40 °C for 1 h, centrifuged at 4025× *g* for 10 min, and the supernatant was precipitated at pH 4.5 for 1 h. The precipitate was then mixed with deionized water and adjusted to pH 7.0 after centrifuging, and freeze-dried to obtain tiger nut protein.

#### 2.3.2. Water and Oil-Holding Capacity

The samples (0.1 g) were mixed with 1 mL of deionized water or soybean oil, with the mass as *m*_1_ and the centrifuge tube mass as *m*_0_, standing at room temperature for 10 min, then centrifuged at 1789× *g* for 10 min to collect the precipitate. The total weight was recorded as *m*_2_. The water-holding capacity (WHC) and oil-holding capacity (OHC) were expressed as grams of water or oil retained per gram of protein and calculated by Equation (4) [29].

(4) $$\mathrm{WHC}/\mathrm{OHC}\;(g/g)=\frac{m_{2}-m_{1}-m_{0}}{m_{1}}$$

#### 2.3.3. Solubility

The samples (20 mg/mL) were mixed thoroughly and shaken for 1 h. The protein content of the supernatant was determined with the Biuret method after centrifuging at 7156× *g* for 5 min at 25 °C. The solubility was calculated by Equation (5).

(5) $$\mathrm{Solubility}\;(\%)=\frac{C_{1}}{C_{2}}\times 100\%$$

where *C*_1_ is the content of protein in the supernatant, *C*_2_ is the content of protein in the original solution.

#### 2.3.4. Foaming Properties

The foaming capacity (FC) and foaming stability (FS) were measured according to the method described by Wang et al. [7] with some modifications. The samples (10 mg/mL) were transferred 20 mL into measuring cylinders and homogenized in a homogenizer (S10, Shanghai Zigui, China) for 2 min. FC and FS were calculated by Equations (6) and (7).

(6) $$\mathrm{FC}\;(\%)=\frac{V_{1}{-V}_{0}}{V_{0}}$$

(7) $$\mathrm{FS}\;(\%)=\frac{V_{1}{-V}_{2}}{V_{1}{-V}_{0}}$$

where *V*_0_ (mL) is the volume of sample before foam, *V*_1_ (mL) and *V*_2_ (mL) are the foam volumes at 0 min and 30 min, respectively.

#### 2.3.5. Emulsifying Properties

The samples (10 mg) were dissolved with 5 mL of deionized water in a tube, and 5 mL of soybean oil was then added, with the height recorded as *H*_0_ (cm). After homogenizing for 30 min, the samples were centrifuged at 1789× *g* for 5 min at 25 °C, and the emulsion layer height was recorded as *H*_1_ (cm). The emulsion layer height of the sample wass treated by heating at 80 °C for 30 min and centrifuging was recorded as *H*_2_ (cm). The emulsibility and emulsion stability were determined by Equations (8) and (9), respectively.

(8) $$\mathrm{Emulsibility}\;(\%)=\frac{H_{1}}{H_{0}}$$

(9) $$\mathrm{Emulsion}\;\mathrm{stability}\;(\%)=\frac{H_{2}}{H_{1}}$$

### 2.4. SDS-PAGE

The SDS-PAGE was performed using a 15% separating gel and 4% stacking gel according to the method described by Sharma et al. [30]. and Liu et al. [27] with modification. The peanut protein, soybean protein, tiger nut protein, and TNP (10 mg/mL) were mixed with the loading buffer (2×) using a vortex (VORTEX-5, Haimen Kylin-Bell Lab, Haimen, China). We used the Bicinchoninicacid (BCA) method to control the protein loads on SDS-polyacrylamide gels between 30 and 40 μg. The molecular mass (MW) marker (10–235 kDa, Shanghai Epizyme Biotech Co., Ltd., Shanghai, China) was utilized in the gel. Electrophoresis was performed with 30 min concentrate at 80 V, and followed by 120 V for 1 h. The log (MW) as a function of Rf (migration distance of the protein/migration distance of the dye front) was plotted to calculate the molecular mass of proteins and TNP.

### 2.5. Antioxidant Activity Measurements

The antioxidant activities of the tiger nut peptides were studied by a combined analysis of DPPH• radical scavenging abilities (RSA), ABTS•^+^ RSA, hydroxyl RSA, and ferric ion-reducing antioxidant power (FRAP). The TNP was configured into a 20 mg/mL solution and diluted to 1, 5, and 10 mg/mL, respectively. The DPPH• RSA measurement was the same as in Section 2.2. The ABTS•^+^ RSA was assayed following the method of Zhu et al. [10]. Their hydroxyl RSA and FRAP were determined using the corresponding kits (Grace Biotechnology Co., Ltd., Suzhou, China). Ascorbic acid (5 mg/mL) was used as the standard. The soybean peptides and peanut peptides were used as the control concentration groups, and we performed the same treatment to evaluate their antioxidant activities.

### 2.6. Amino Acid Analysis

Amino acid analysis was performed using an automated amino acid analyzer (LA8080, Hitachi High-Tech Co., Ltd., Tokyo, Japan). The samples were weighed, hydrolyzed with hydrochloric acid, fixed, and passed through a 0.22 μm microporous filter membrane.

### 2.7. Isolation and Purification of TNP

TNP was isolated and purified according to the previous study [16]. The TNP was configured into a 50 mg/mL solution and dialyzed for 24 h using a dialysis bag with a cutoff molecular weight of 100 Da. The treated peptide solution was filtered through a 0.8 μm microporous membrane filtration to remove remaining particles and contaminants. Three ultrafiltration membranes of the ultrafiltration centrifuge tube with molecular weight cutoffs of 1, 3, and 10 kDa (Millipore, USA) were used to divide the solution into four fractions, >10 kDa (TNP-1), 3–10 kDa (TNP-2), 1–3 kDa (TNP-3), and <1 kDa (TNP-4), by centrifuging at 1789× *g* for 30 min at 25 °C. The empty column (2.6 cm × 70 cm, Ruida Henghui, Beijing, China) was filled with Sephadex G-15 (HA-0220, Ruida Henghui, Beijing, China) by using the wet–natural sedimentation method. The peptides were dissolved, and the column was equilibrated with ultrapure water. The flow rate was maintained at 1.5 mL/min. The absorbance was measured at 220 nm, and the elution peaks were collected for antioxidant activity measurement.

### 2.8. Cytoprotective Effects on HepG2 and Caco-2 Cells Damage Induced by H_2_O_2_

Fetal bovine serum (FBS, 10% [*v*/*v*]), penicillin–streptomycin (1% [*v*/*v*]), and MEM were the ingredients of the culture medium used to produce HepG2 cells; Caco-2 cells needed 20% FBS. The culture conditions were maintained at 5% CO_2_ and 37 °C.

#### 2.8.1. Cytotoxicity Assay

The cell density was kept at 4.5 × 10^3^ cells/well in the 96-well plate and cultured until it was adherent. The supernatant was then removed, and 100 µL of 0.1, 0.2, 0.3, 0.4, and 0.5 mg/mL samples were added for another 24 h cultivation, respectively. Cell viability was measured by MTT solution. The 100 µL DMSO was added after removing the supernatant, and the absorption of the sample was measured at 490 nm to determine the cytotoxicity of the peptides [31].

#### 2.8.2. H_2_O_2_-Induced Oxidative Stress Assay and Indicator Measurement

The cell culture process was the same as Section 2.8.1. A total of 100 μL of 0.2, 0.5, 1, 1.2, and 1.5 mM H_2_O_2_ was added into the cells for 2 h, respectively, to establish the oxidative damage model with the cell viability around 50%. The cells were preincubated with the peptide samples for 24 h before adding H_2_O_2_ to investigate their cytoprotective effects. The ROS (Shanghai Beyotime Biotech. Inc., Shanghai, China), SOD, CAT, GSH-Px, MDA, and ATP content (Nanjing Jiancheng Co., Nanjing, China) were determined by kits detection.

#### 2.8.3. Microscopic Observation of Morphological Change

The cell morphology was observed by an inverted biomicroscope (DSZ2000X, Beijing Cnmicro Hengye, Beijing, China) at 10× magnification.

### 2.9. Statistical Analysis

All the experiments were measured three times, and the data were expressed as mean ± standard deviation (SD). Analysis of variance (ANOVA) by Duncan’s multiple tests (*p* < 0.05) was conducted via SPSS 26.0 software (SPSS Inc., Chicago, IL, USA).

## 3. Results

### 3.1. Preparation of the TNP

The DH and DPPH• RSA of TNP were identified as 52.33 ± 1.09% and 75.23 ± 0.67%, respectively, by the optimized enzymolysis with 10,000 U/g of enzyme/substrate ratio, 55 °C of hydrolysis temperature, and 3 h of hydrolysis time. The properties of the obtained TNP were further studied.

### 3.2. Basic Functional Properties Research Results

#### 3.2.1. Water/Oil-Holding Capacity and Solubility

The water/oil-holding capacity and solubility of tiger nut protein and TNP are summarized in Table 1. The WHC and OHC of protein and peptide are related to functional properties, texture, mouthfeel, and the storage stability of the food system. After enzymatic hydrolysis, the TNP presented decreased WHC and OHC, and dramatically increased solubility compared with those of the tiger nut protein. This was consistent with the fact that decreased WHC and OHC were considered higher solubility. This could be because enzymatic hydrolysis broke down the peptide chain in the protein structure, resulting in smaller molecular weight and carboxyl and exposed amino-terminal groups. The peptides produced became more hydrophilic, and the solubility increased, with decreased water and oil absorption. The peptides have a good solubility and mass loss upon disposal, resulting in a negative value.

#### 3.2.2. Foaming and Emulsifying Properties

The foaming and emulsifying properties are essential functional properties of protein and peptide products used in food industry applications. The FC, FS, emulsibility, and emulsion stability of tiger nut protein and TNP are shown in Table 1. Good solubility is a critical factor for excellent foam properties [32]. The TNP performed higher FS compared with the tiger nut protein. There was a lower value in the viscosity of TNP and a rise in exposure of hydrophobic groups of the system caused by protein hydrolysis, which could be responsible for the decreased FC. Exposure to hydrophobic groups could lead to an increase in insoluble proteins or peptides, and the insoluble proteins and peptides were adsorbed to the interface, increasing the thickness of the interface layer due to protein–protein interaction. The insoluble protein particles played a beneficial role in FS properties [33].

Proteins and peptides are characterized by presenting hydrophilic and hydrophobic functional groups. Therefore, they can adsorb oil at the oil–water interface to form emulsions and ensure their stability without flocculation for some time [34]. The TNP performed inferior emulsifying performance compared with protein, with decreased emulsibility and emulsion stability, as shown in Table 1. The lower emulsification performance may be attributed to the small molecular weight of TNP, and the formed adsorption film was not enough to maintain oil–water stability, thus decreasing the emulsion stability.

### 3.3. Antioxidant Activity Analysis

#### 3.3.1. Antioxidant Activity of Crude Peptides

**Figure 1:**
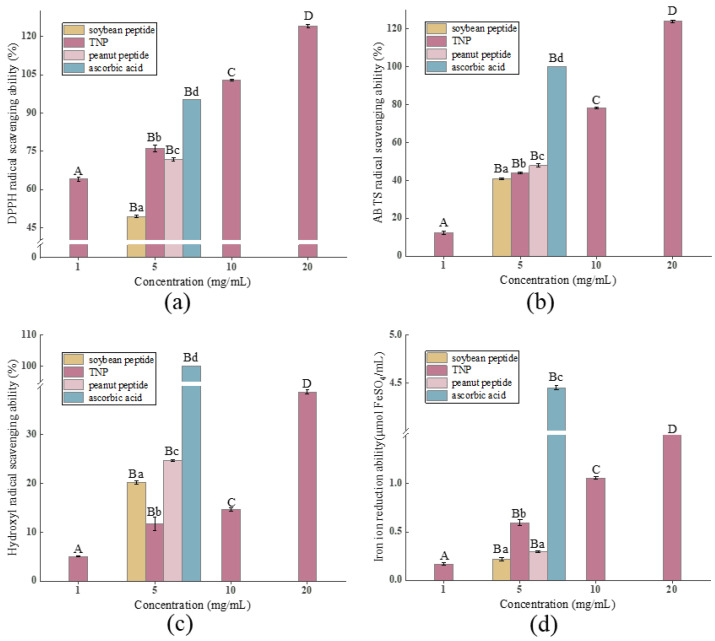
The DPPH• radical scavenging activity (**a**), ABTS•^+^ radical scavenging activity (**b**), hydroxyl radical scavenging activity (**c**), and FRAP (**d**) of peptides of antioxidant properties of TNP, soybean peptides, and peanut peptides at the concentrations of 1–20 mg/mL. Different uppercase letters and lowercase letters represent statistically significant differences (*p* < 0.05).

The antioxidant activities of the peptides were determined by measuring DPPH• RSA, ABTS•^+^ RSA, hydroxyl RSA, and FRAP in this study (Figure 1), and their amino acid composition was summarized in Table 2. The DPPH• RSA, ABTS•^+^ RSA, hydroxyl RSA, and FRAP of TNP increased with concentration increasing, consistent with the trend for camel bone peptides reported by Wang et al. [7]. It is widely accepted that the free radical DPPH• can be transform into stable molecule DPPH-H when absorbing an electron or a proton from compounds such as antioxidants [35], and antioxidants can also inhibit the formation of blue-green ABTS•^+^, causing the color to fade. The DPPH• radical scavenging rate of TNP was 64.05–124.07% over a concentration range of 1–20 mg/mL, and it reached 76.16 ± 1.33% at 5 mg/mL, significantly greater than that of soybean peptides (49.43 ± 0.51%) and peanut peptides (71.89 ± 0.60%) (*p* < 0.05). Additionally, the ABTS•^+^ radical scavenging rate of TNP reached 44.00 ± 0.49% at 5 mg/mL, more than that of soybean peptides (47.80 ± 0.88%) (*p* < 0.05). These indicated that the TNP is effective in scavenging both DPPH• and ABTS•^+^ free radicals, meaning it could provide many electrons.

The hydroxyl RSA of TNP was 5.04–38.82% at 1–20 mg/mL and exhibited a low value of 11.71 ± 1.31% at 5 mg/mL compared with other peptides. This showed relatively poor ability of TNP to convert hydroxyl radicals and stop the radical chain reaction. It was widely acknowledged that the presence of acidic AAs such as Glu and Asp contributed to strong capacity of peptides to scavenge hydroxyl radicals. And the lower hydroxyl RSA of TNP presented could be supported by their AAs analysis results with low content of the acidic AAs (Table 2).

Antioxidants can also reduce Fe^3+^-tripyridyltriacridazine (Fe^3+^-TPTZ) to produce Fe^2+^-TPTZ (blue color). The FRAP of TNP was 0.17–1.78 at 1–20 mg/mL. It reached 0.60 ± 0.03 at 5 mg/mL, higher than that of soybean peptides (0.22 ± 0.02) and peanut peptides (0.29 ± 0.01) (*p* < 0.05). Some sulfur-containing AAs were significant contributors to FRAP, so it may be possible that the high FRAP value due to the sulfur-containing amino acid (such as Met), because its sulfhydryl group is prone to oxidation by reactive species (Table 2) [36]. These indicated that the peptides from tiger nut (TNP) had higher antioxidant activities with strong DPPH• and ABTS•^+^ RSA and FRAP than soybean and/or peanut antioxidant peptides.

The amino acid composition of peptide plays an important role in its antioxidant activity. The antioxidant activity of protein hydrolysates depends on the contents of hydrophobic AAs [37]. This is because the hydrophobicity of the antioxidant peptides allows them to readily interface hydrophobic interactions with the lipid bilayer of the membrane and enter the target organ, thereby exhibiting excellent free radical scavenging properties [38]. The functional properties of proteins are often closely related to their amino acid composition, so the amino acid composition represents the potential quality of plant proteins [39]. It was observed in the amino acid composition results that TNP had high hydrophobic amino acid content, particularly Ile, Ala, Met, Val, Pro, and Leu (Table 2), supporting the higher antioxidant indexes of TNP compared with the soybean and/or peanut antioxidant peptides. In conclusion, these results indicated that the peptides obtained from tiger nut had strong antioxidant activities, considered a potential antioxidant peptide.

#### 3.3.2. Antioxidant Activity of TNPs by Isolation and Purification

**Figure 2:**
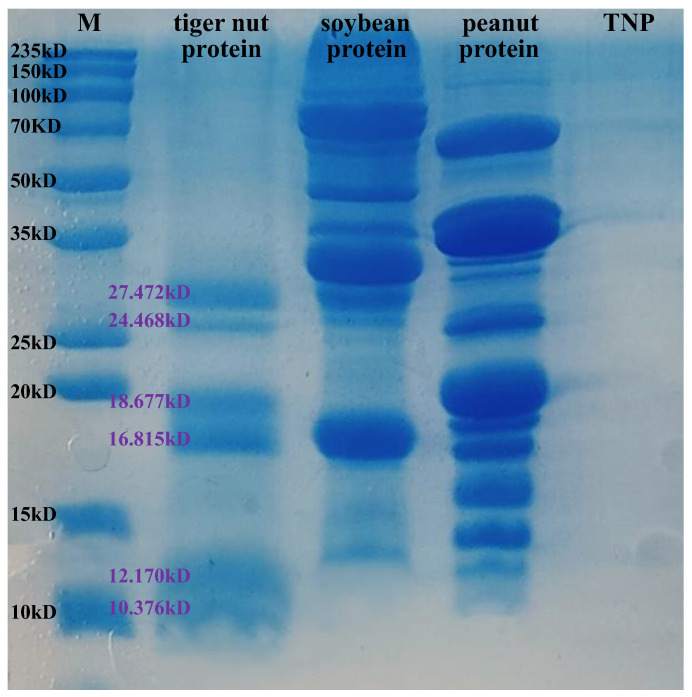
SDS-PAGE of proteins and tiger nut peptides (TNP). The purple letters represent the molecular weight of tiger nut protein.

The SDS-PAGE profiles of tiger nut protein, soybean protein, peanut protein, and TNP are depicted in Figure 2. As shown in the profiles, the tiger nut protein had a smaller molecular weight compared with the soybean and peanut proteins. The tiger nut protein contained six main bands distributed at 27.472 kD, 24.468 kD, 18.677 kD, 16.815 kD, 12.170 kD, and 10.376 kD molecular weights. Accordingly, it was expected that there might be a low molecular weight structure less than 10 kD for TNP, but it was difficult to separate in 15% polyacrylamide gel and the SDS-PAGE system. According to the previous experiment [16], four fractions with different molecular weights were obtained after ultrafiltration: TNP-1 (>10 kD), TNP-2 (3–10 kD), TNP-3 (1–3 kD), and TNP-4 (<1 kD). The DPPH• RSA, ABTS•^+^ RSA, hydroxyl RSA, and FRAP of the peptides increased with the molecular weight decreasing, showing stronger antioxidant capacity. Small peptides with lower molecular weight tend to have great potential for bioactivity [18,40].

**Figure 3:**
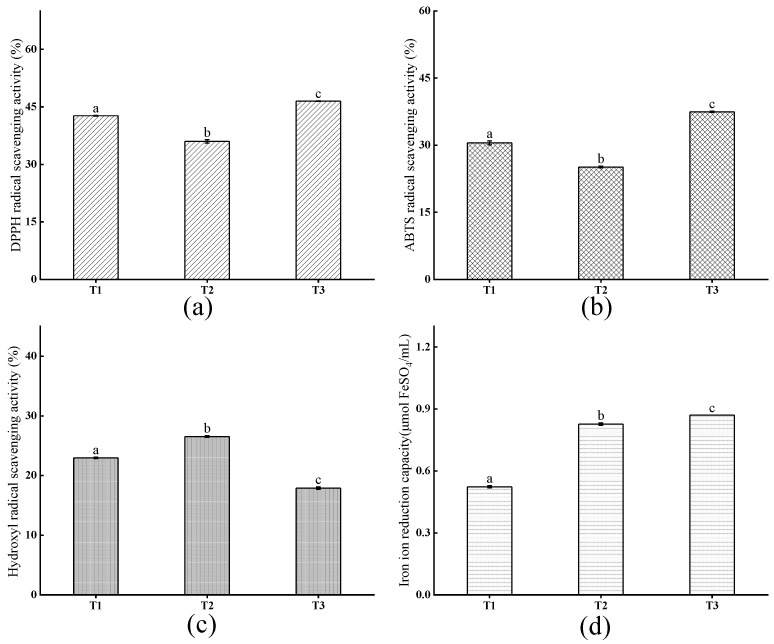
The DPPH• radical scavenging activity (**a**), ABTS•^+^ radical scavenging activity (**b**), hydroxyl radical scavenging activity (**c**), and FRAP (**d**) of T1, T2, and T3 at the concentration of 0.5 mg/mL. T1, T2, and T3 represent three elution peaks. Different letters represent statistically significant differences (*p* < 0.05).

The TNP-4 was separated into three fractions (T1, T2, T3) by a Sephadex G-15 gel column with elution time increasing. As shown in Figure 3, the T3 exhibited the highest DPPH• radical scavenging rate, ABTS•^+^ radical scavenging rate, and FRAP with the values of 46.49 ± 0.07%, 37.45 ± 0.15%, and 0.87 ± 0.00 at 0.5 mg/mL, respectively, suggesting strong antioxidant capacities. However, T2 has the highest hydroxyl radical scavenging rate of 26.53 ± 0.14%, and P6 (FLDPLIPPI), which was previously identified in T2 [16], also showing excellent hydroxyl RSA at 1 mg/mL. However, a P1 (QPPSQPQPML) component with excellent antioxidant capacity was detected in T1, which was not well reflected in T1, which was speculated to be due to the antagonism between various components in the peptide solution of T1, and the identified peptide segment may contain errors after bioinformatics analysis.

### 3.4. Cytoprotective Functions on HepG2 and Caco-2 Cells

#### 3.4.1. Cell Cytotoxicity

Cell experiments provide a promising alternative apart from the in vitro chemical technique to evaluate antioxidant activity. The cytotoxic effect of TNP, TNP-4, T1, T2, and T3 on HepG2 cells and Caco-2 cells were shown in Figure 3, respectively. All the viabilities of the tiger nut peptide-treated HepG2 and Caco-2 cells increased, and they presented dose dependence, showing increased viabilities with the augmentation of the TNPs at 0.1–0.5 mg/mL. These indicated that the TNPs were non-toxic to HepG2 and Caco-2 cells between 0.1 and 0.5 mg/mL, and promoted their proliferation to some extent. In addition, the cell viability of the T1, T2, and T3 groups was significantly greater than GSH (*p* < 0.05). In particular, the cell group treated by T3 had the highest cell viability among the samples of TNP, TNP-4, T1, T2, and T3, reaching 152.87 ± 1.74% for HepG2 cells and 139.82 ± 3.60% for Caco-2 cells at 0.5 mg/mL, efficiently promoting cell proliferation. In previous experiments, P1 (QPPSQPQPML) and P4 (FLDPLIPPI) components also significantly promoted cell proliferation at 0.5 mg/mL [16].

#### 3.4.2. Cytoprotective Effects on Cell Damage Induced by H_2_O_2_

##### Protection of TNPs Against Oxidative Stress in Cells

**Figure 4:**
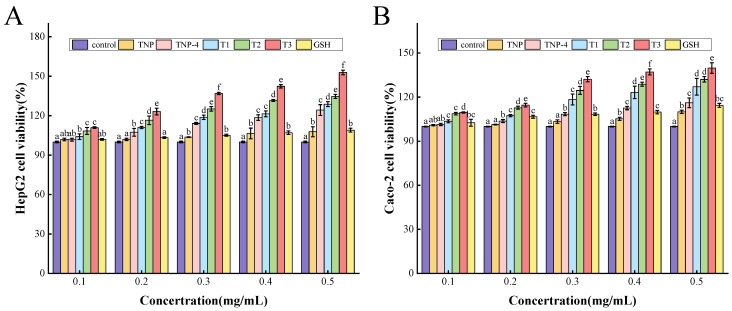
The cytotoxic effect of TNP, TNP-4, T1, T2, and T3 on HepG2 cells (**A**) and Caco-2 cells (**B**). Different letters represent significant differences between different samples in the same group (*p* < 0.05).

The cytoprotective potentials of TNPs were further demonstrated by testing the viability of HepG2 and Caco-2 cells cultured in various H_2_O_2_ concentrations. The results showed that the cell viability reduced to 52.05 ± 2.33% and 52.29 ± 1.15% for HepG2 and Caco-2 cells, respectively, after treatment with 1 mM H_2_O_2_ for 2 h, which were close to 50%. And it was indicated that the cell viability of HepG2 cells and Caco-2 cells damage groups were 50.99 ± 2.02% and 50.49 ± 1.37%, respectively (Figure 4A,B). The oxidative damage model induced by H_2_O_2_ was successfully established.

**Figure 5:**
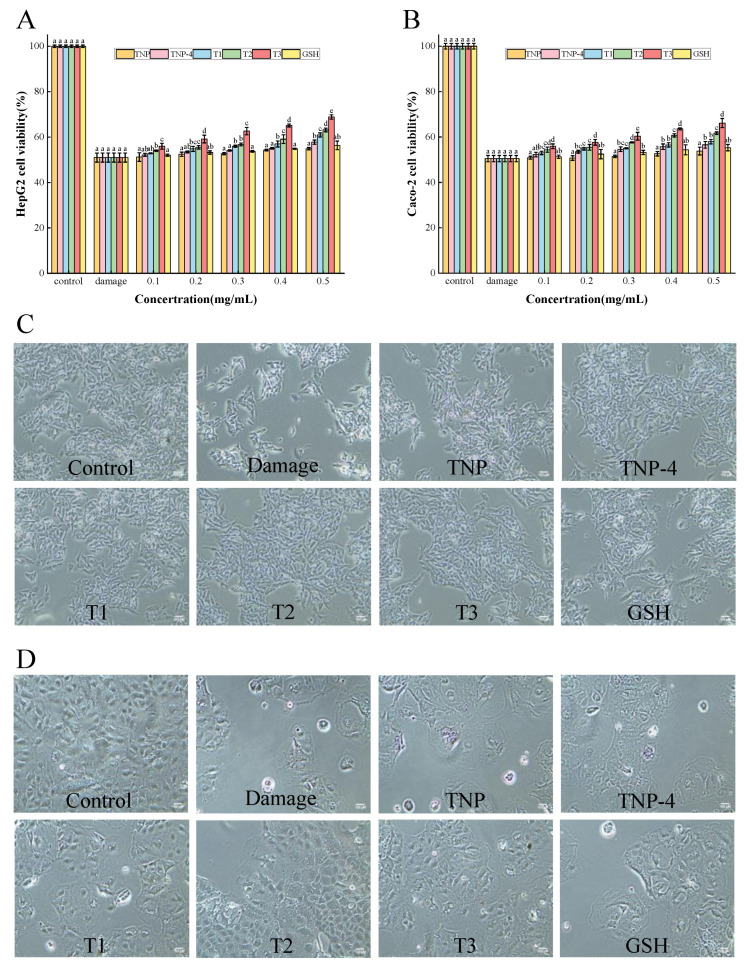
Effect of TNPs on cell viability of HepG2 cells (**A**) and Caco-2 cells (**B**) in H_2_O_2_-induced oxidative stress with the treatment of TNP, TNP-4, T1, T2, T3, and GSH, and their morphological changes ((**C**), HepG2 cells; (**D**), Caco-2 cells). The control group means normally growing cells. The damage group means H_2_O_2_-induced cells. The remaining groups were treated with peptides at 0.5 mg/mL and induced by H_2_O_2_, Different letters represent significant differences between different samples in the same group (*p* < 0.05).

As shown in Figure 5A,B, the cell viability of the tiger nut peptide-treated groups, (especially TNP-4, T1, T2, and T3) increased compared to the damage groups. This demonstrated that the peptides obtained from tiger nut could protect the HepG2 and Caco-2 cells against oxidative stress, and the cytoprotective effect enhanced with peptide concentration increasing, showing a dose-dependent pattern. The TNP had the weakest protective effect among the tiger nut peptide samples, similar to GSH. After ultrafiltration, gel separation, and purification, the purity of peptides increased, accompanied by decreased molecular weight, and the protective effect of peptide samples on the cells enhanced. This trend was consistent with the results of cytotoxicity assay in Figure 4. And the T3 exhibited the strongest cytoprotective effect, significantly higher than that of GSH, TNP, TNP-4, T1, and T2 (*p* < 0.05), increasing the viability of HepG2 cells and Caco-2 cells by 17.87% and 15.67% at 0.5 mg/mL, respectively. The protective effect of TNPs (TNP-4, T1, T2, T3) against oxidative damage was more pronounced than that of GSH, probably as a consequence of the difficulty of GSH in crossing the cell membrane [41]. It is worth mentioning that P1 (QPPSQPQPML) and P4 (FLDPLIPPI) also exhibited better cytoprotective effects than GSH. These results suggested that the peptides TNP-4, T1, T2, T3 obtained from tiger nut could adequately protect cells against oxidative damage.

##### Morphological Observation

The morphology of H_2_O_2_-induced HepG2 cells and Caco-2 cells models treated by TNP, TNP-4, T1, T2, T3, and GSH was presented in Figure 5C,D. The damage group showed few surviving cells by H_2_O_2_, and most of the cells disintegrated and floated in the medium. However, it was obvious that the peptides from tiger nut and GSH efficiently protected the HepG2 cells and Caco-2 cells against H_2_O_2_-stimulated oxidative damage. Particularly, the cells by TNP-4, T1, T2, and T3 showed low apoptosis, and presented similar morphology to those in the control group including clear cell outlines and similar shapes and sizes, suggesting an excellent cytoprotective effect of the peptides. These were consistent with their protective effect results of cellular oxidative stress.

##### CAT, SOD, GSH-PX Activities, and MDA, ATP, ROS Contents

H_2_O_2_ is a common inducer of oxidative stress and can cause damage through the Fenton reaction [42]. The generation of hydroxyl radicals and hydroxyl ions by H_2_O_2_ has been demonstrated to directly attack cell membranes, proteins, and DNA, and these damages can cause cell dysfunction and death. MDA, a lipid peroxidation product, can indirectly assist in demonstrating the degree of oxidative damage triggered by ROS. Previous research has demonstrated that intracellular SOD, GSH-PX, and CAT activities can be enhanced by bioactive peptides derived from food-sourced proteins, hence providing cell protection [40,43], with increased enzyme activity, increased cellular activity, and increased energy production. Consequently, the present study sought to evaluate cellular ROS levels, SOD, GSH-PX, and CAT activities, and MDA and ATP contents.

**Figure 6:**
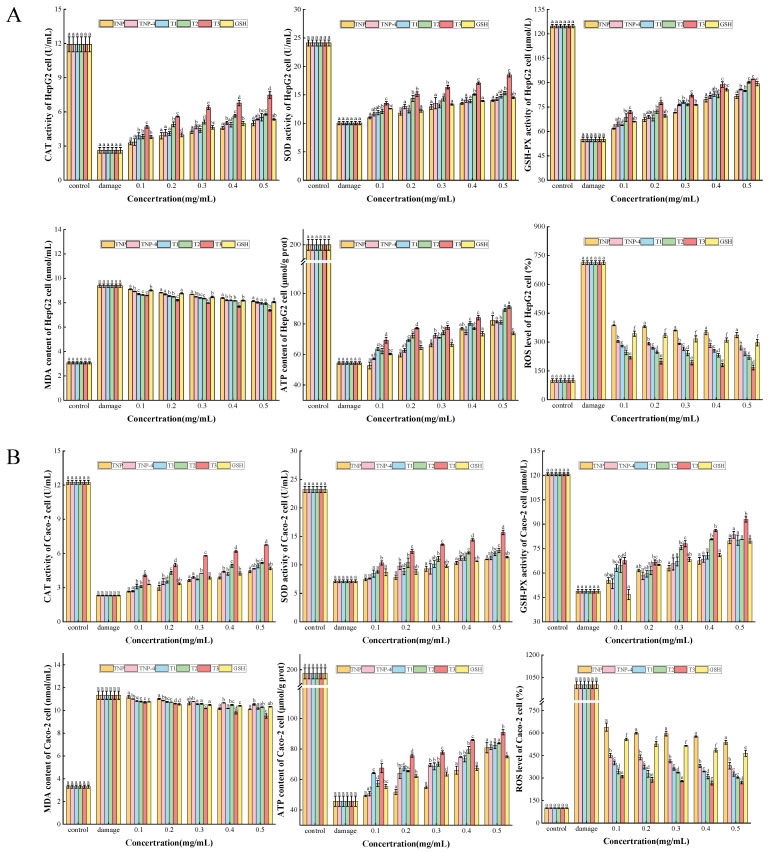
The cellular antioxidant activity analysis containing CAT, SOD, GSH-PX activities and MDA, ATP, ROS contents of H_2_O_2_-oxidized HepG2 cells (**A**) and Caco-2 cells (**B**) with the treatment of TNP, TNP-4, T1, T2, T3, and GSH. Different letters represent significant differences between different samples in the same group (*p* < 0.05).

As illustrated in Figure 6, the activity of HepG2 and Caco-2 cells decreased to 50.98 ± 2.02% and 50.49 ± 1.3%, respectively, in comparison to the control group. The activities of SOD, GSH-PX, and CAT were significantly reduced after treatment with 1 mM H_2_O_2_ for 2 h, the ROS level and MDA content were significantly increased (*p* < 0.05). This suggested that the cells were severely damaged due to intense oxidation by H_2_O_2_. In comparison to the damage group, after preincubation of the cells with 0.1–0.5 mg/mL TNPs and GSH for 24 h and then after it was oxidatively damaged, the enzyme activities of cells increased gradually, showing a dose-dependent pattern, while ROS level and MDA content decreased gradually. These intracellular oxidative stress indicators correspond to cell viability, indicating that cell viability is regulated by these endogenous antioxidant enzymes. The phenomenon wherein T1, T2 and T3 outperformed TNP-4 confirmed the characteristics of G-15 purification. These results implied that the peptides from tiger nut, especially T3, have excellent protective effects on both cells, and could perform cytoprotective effects against oxidative damage by enhancing intracellular enzyme activity and lowering oxidative stress levels.

## 4. Conclusions

In this study, antioxidant peptides were obtained from tiger nut by Alcalase. The functional properties, in vitro antioxidant activity, and their chemical antioxidant activity and cytoprotective functions on H_2_O_2_-mediated oxidative-injured HepG2 and Caco-2 cells were systematically investigated. The results showed that the TNP has strong antioxidant activity compared to traditional soybean and peanut peptides. The antioxidant activity of the peptides was improved greatly by gradual ultrafiltration and fractionation, showing a negative correlation with their molecular weight. Among the TNPs, the T3 fraction demonstrated excellent antioxidant capacity at both the in vitro and cellular levels, displaying strong DPPH• RSA and FRAP, and promoting and protective effects on H_2_O_2_-injured HepG2 and Caco-2 cells by augmenting endogenous antioxidant enzyme activities and reducing oxidative stress levels. These findings provide insights into the peptides from tiger nuts. Animal experiments can be conducted to further verify the antioxidant properties of TNP in future studies. Tiger nut can be considered an efficient source of antioxidant peptides in the food industry, and related tiger nut-based health food can be further developed.

## Figures and Tables

**Table 1:** Water/oil-holding capacity, solubility, and foaming and emulsifying properties of tiger nut protein and TNP.

Function	Tiger Nut Protein	TNP
WHC (g/g)	2.86 ± 0.01 ^a^	—
OHC (g/g)	4.18 ± 0.01 ^a^	1.99 ± 0.01 ^b^
Solubility (%)	6.08 ± 0.12 ^a^	31.92 ± 2.25 ^b^
FC (%)	31.67 ± 2.89 ^a^	15.00 ± 0.00 ^b^
FS (%)	63.49 ± 5.50 ^a^	80.00 ± 0.00 ^b^
Emulsibility (%)	50.98 ± 0.00 ^a^	41.32 ± 0.00 ^b^
Emulsion stability (%)	76.92 ± 0.01 ^a^	60.00 ± 0.00 ^b^

Different letters in the same group indicate significant differences (*p* < 0.05). WHC, water-holding capacity; OHC, oil-holding capacity; FC, foaming capacity; FS, foaming stability.

**Table 2:** Amino acid analysis of proteins and TNP.

Amino Acids (g/100 g)	Tiger Nut Protein	TNP	Soybean Peptides	Peanut Peptides
Aspartic (Asp)	6.62 ± 0.08 ^a^	9.02 ± 0.13 ^b^	12.17 ± 0.29 ^d^	12.71 ± 0.17 ^c^
Threonine (Thr)	4.82 ± 0.16 ^a^	4.65 ± 0.26 ^a^	3.99 ± 0.18 ^c^	2.59 ± 0.13 ^b^
Serine (Ser)	4.38 ± 0.40 ^a^	4.05 ± 0.45 ^a^	4.22 ± 0.86 ^a^	4.30 ± 0.65 ^a^
Glutamic (Glu)	18.48 ± 0.91 ^a^	17.40 ± 0.64 ^a^	18.97 ± 0.70 ^ab^	20.73 ± 0.75 ^b^
Glycine (Gly)	5.75 ± 0.04 ^a^	3.05 ± 0.06 ^b^	4.29 ± 0.25 ^c^	6.08 ± 0.13 ^a^
Alanine (Ala)	5.61 ± 0.08 ^a^	7.47 ± 0.13 ^b^	4.67 ± 0.25 ^c^	4.26 ± 0.16 ^c^
Cysteine (Cys)	1.54 ± 0.12 ^a^	0.86 ± 0.06 ^b^	1.16 ± 0.11 ^bc^	1.23 ± 0.17 ^ac^
Valine (Val)	6.06 ± 0.24 ^a^	6.74 ± 0.39 ^a^	5.10 ± 0.25 ^c^	4.21 ± 0.23 ^b^
Methionine (Met)	1.65 ± 0.04 ^a^	2.00 ± 0.00 ^a^	1.12 ± 0.23 ^b^	0.98 ± 0.29 ^b^
Isoleucine (Ile)	4.52 ± 0.52 ^a^	4.74 ± 0.26 ^a^	4.71 ± 0.27 ^a^	3.54 ± 0.20 ^b^
Leucine (Leu)	8.44 ± 0.04 ^a^	8.47 ± 0.00 ^a^	8.11 ± 0.20 ^a^	7.02 ± 0.19 ^b^
Tyrosine (Tyr)	2.36 ± 0.16 ^a^	2.46 ± 0.26 ^a^	3.36 ± 0.14 ^b^	3.42 ± 0.04 ^b^
Phenylalanine (Phe)	4.01 ± 0.12 ^a^	4.56 ± 0.39 ^a^	5.31 ± 0.12 ^b^	5.48 ± 0.09 ^b^
Lysine (Lys)	6.34 ± 0.08 ^a^	4.51 ± 0.06 ^b^	6.78 ± 0.12 ^d^	3.72 ± 0.03 ^c^
Histidine (His)	3.42 ± 0.08 ^a^	2.78 ± 0.06 ^b^	2.88 ± 0.14 ^b^	2.44 ± 0.01 ^c^
Arginine (Arg)	8.25 ± 0.16 ^a^	10.30 ± 0.26 ^b^	7.61 ± 0.02 ^d^	12.63 ± 0.06 ^c^
Proline (Pro)	7.74 ± 0.32 ^a^	6.93 ± 0.26 ^b^	5.57 ± 0.05 ^d^	4.64 ± 0.03 ^c^
Acidic amino acids (NCAA)	25.1 ± 0.99 ^a^	26.42 ± 0.77 ^a^	33.44 ± 0.58 ^b^	31.13 ± 0.41 ^c^
Basic Amino Acids (PCA)	18.01 ± 0.32 ^a^	17.59 ± 0.26 ^ab^	18.79 ± 0.01 ^c^	17.26 ± 0.25 ^b^
Hydrophobic amino acids (HAA)	39.58 ± 0.99 ^a^	41.77 ± 1.36 ^a^	31.35 ± 0.64 ^b^	35.75 ± 0.66 ^c^

Different letters in the same amino acid group indicate significant differences (*p* < 0.05).

## Data Availability

The original contributions presented in the study are included in the article; further inquiries can be directed to the corresponding author.

## Abbreviations

The following abbreviations are used in this manuscript:

TNP	tiger nut peptide
ROS	reactive oxygen species
H_2_O_2_	hydrogen peroxide
DH	degree of hydrolysis
pI	isoelectric point
DPPH•	1,1-diphenyl-2-picroyl
ABTS•^+^	2,2-azino-bis-3-ethylbenzothiazoline-6-sulfonic acid
•OH	hydroxyl radical
FRAP	hiazoline-6-sulfonic acid
RSA	radical scavenging ability
WHC	water-holding capacity
OHC	oil-holding capacity
FC	foaming capacity
FS	foaming stability
SDS-PAGE	sodium dodecyl sulphate polyacrylamide gel electrophoresis
MDA	malondialdehyde
SOD	superoxide dismutase
CAT	catalase
GSH	glutathione
GSH-PX	glutathione peroxidase
ATP	adenosine triphosphate
MEM	minimum essential medium
FBS	fetal bovine serum
MTT	3- (4,5-dimethyl-2-thiazolyl)-2,5-diphenyl-2-H-tetrazolium bromide
DMSO	dimethyl sulfoxide
AA	amino acid
